# Supplementary material for: Leveraging pleiotropy for joint analysis of genome-wide association studies with per trait interpretations
Source: PLoS Genet. 2022 Nov 7;18(11):e1010447. doi: 10.1371/journal.pgen.1010447 (PMC9671458; doi:10.1371/journal.pgen.1010447)
Supplement: S1 Text — (PDF) [file pgen.1010447.s001.pdf]

Supplementary Note: Leveraging pleiotropy for joint analysis of genome-wide association studies with per trait interpretations

Kodi Taraszka[1] Noah Zaitlen[2,3] Eleazar Eskin[1,3,4*]

[1]Department of Computer Science, University of California, Los Angeles, California, 90095, United States

[2]Department of Neurology, University of California, Los Angeles, California, United States

[3] Department of Computational Medicine, University of California, Los Angeles, California, United States

[4]Department of Human Genetics, University of California, Los Angeles, California, United States

∗ Email corresponding author at: eeskin@cs.ucla.edu

UK Biobank Data

Trait	Genetic Variance	Sample Size	LD-Score Intercept
body mass index ($\mathcal{B}$)	0.241	336,107	1.082
diastolic blood pressure ($\mathcal{D}$)	0.129	317,756	1.074
height ($\mathcal{H}$)	0.429	336,574	1.237
systolic blood pressure ($\mathcal{S}$)	0.114	317,754	1.108

Table A. The genetic variance and sample sizes from the 2017 UK Biobank release. We used summary statistics for body mass index, diastolic blood pressure, height, and systolic blood pressure from the 2017 release of the UK Biobank as input for simulations. We reported the sample sizes, genetic variance estimated by LD-Score regression, and the LD-Score intercept.

We used four traits from the UK Biobank released in 2017 and in 2018 as the basis of our simulations [1, 2]. For both sets of summary statistics, only the variants which were biallelic, have non-ambiguous strands, a minor allele frequency greater than 1%, an INFO score greater than 80%, and found in the 1000 Genomes European reference panel were retained [3]. We used LD-Score regression [4] to calculate the genetic variance of each trait as shown in Table A and Table C. For calculating genetic covariance and environmental covariance, we used cross-trait LD-Score regression [5]. The genetic covariance produced by the software was reported in Table B and Table D. By taking the intercept and scaling it by $\frac{\sqrt{N_1 N_2}}{N_s}$ where N_1 is the sample size in trait 1, N_2 is the sample size in trait 2, and N_s is the number of overlapping individuals, we were able to recover the phenotypic covariance. By subtracting the genetic covariance from the phenotypic covariance, the environmental covariance is estimated. As we used summary statistics and the true sample overlap was unknown, we assumed there were no trait specific missing individuals, and we, therefore, set $N_s = min\{N_1, N_2\}$.

Trait 1	Trait 2	Genetic Correlation	Environmental Correlation	LD-Score Intercept
body mass index ($\mathcal{B}$)	diastolic blood pressure ($\mathcal{D}$)	0.305	0.258	0.303
body mass index ($\mathcal{B}$)	height ($\mathcal{H}$)	-0.165	-0.084	-0.137
body mass index ($\mathcal{B}$)	systolic blood pressure ($\mathcal{S}$)	0.166	0.198	0.219
diastolic blood pressure ($\mathcal{D}$)	height ($\mathcal{H}$)	-0.125	-0.004	-0.025
diastolic blood pressure ($\mathcal{D}$)	systolic blood pressure ($\mathcal{S}$)	0.648	0.686	0.765
height ($\mathcal{H}$)	systolic blood pressure ($\mathcal{S}$)	-0.144	-0.104	-0.132

Table B. The genetic and environmental correlation used from the 2017 version of UK Biobank data. We used summary statistics for body mass index, diastolic blood pressure, height, and systolic blood pressure from the 2017 release of the UK Biobank as input to simulations. We used cross-trait LD-Score regression to estimate the genetic and environmental correlation and report the LD-Score intercept.

Trait	Genetic Variance	Sample Size	LD-Score Intercept
body mass index ($\mathcal{B}$)	0.243	359,983	1.057
diastolic blood pressure ($\mathcal{D}$)	0.132	340,162	1.068
height ($\mathcal{H}$)	0.469	360,388	1.270
systolic blood pressure ($\mathcal{S}$)	0.139	340,159	1.070

Table C. The genetic variance and sample sizes used in simulations and real data analyses. Using the summary statistics for body mass index, diastolic blood pressure, height, and systolic blood pressure from the 2018 release of the UK Biobank, we estimated the genetic variance with LD-Score regression. We report the sample sizes, genetic variance, and LD-Score intercept.

Trait 1	Trait 2	Genetic Correlation	Environmental Correlation	LD-Score Intercept
body mass index ($\mathcal{B}$)	diastolic blood pressure ($\mathcal{D}$)	0.305	0.228	0.275
body mass index ($\mathcal{B}$)	height ($\mathcal{H}$)	-0.164	-0.066	-0.121
body mass index ($\mathcal{B}$)	systolic blood pressure ($\mathcal{S}$)	0.174	0.15	0.177
diastolic blood pressure ($\mathcal{D}$)	height ($\mathcal{H}$)	-0.122	-0.001	-0.030
diastolic blood pressure ($\mathcal{D}$)	systolic blood pressure ($\mathcal{S}$)	0.663	0.678	0.768
height ($\mathcal{H}$)	systolic blood pressure ($\mathcal{S}$)	-0.151	-0.047	-0.083

Table D. The genetic and environmental correlation used in real data analyses and simulations. We used summary statistics for body mass index, diastolic blood pressure, height, and systolic blood pressure from the 2018 release of the UK Biobank as input to cross-trait LD-Score regression. We report the genetic and environmental correlation as well as the LD-Score intercept.

For simulations, PAT used the reported values to define its likelihood ratio test; MI GWAS, however makes no assumptions about the phenotypic covariance. HIPO and MTAG defined their parameters slightly differently, but all methods used the same results from LD-Score regression and cross-trait LD-Score regression. For simulations comparing the power between MI GWAS and PAT, we set the environmental correlation to 0% for all traits. Simulations comparing PAT to MI GWAS and for showing the stability of null simulations used the 2017 version of the UK Biobank summary statistics (see Table A and Table B for values). All other simulations and real data analyses were

based on the summary statistics from 2018 (see Table C and Table D). The switch in summary statistic version was due to the 2017 version of the UK Biobank results being no longer available which prevented reproduction of results.

Assessing the impact of environmental correlation on PAT's power

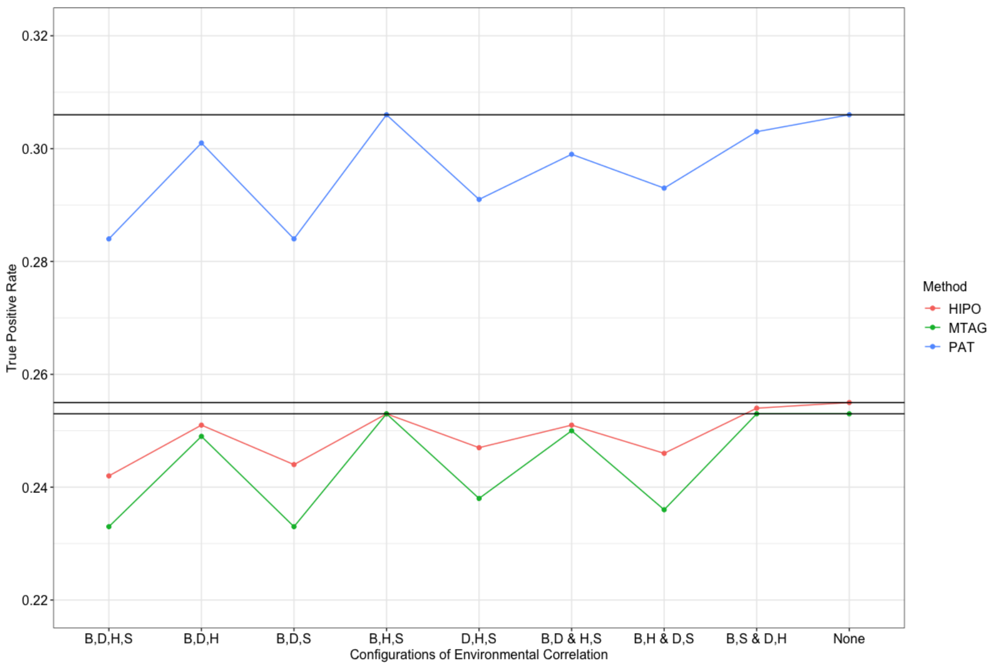

Fig A. Comparison of multi-trait GWAS methods. We simulated nine configurations of one million variants with varying environmental correlation between traits. The x-axis corresponds to the list of configurations while the y-axis shows the true positive rate of each method. We compared three methods, PAT (in blue), MTAG (in green) and HIPO (in red) and included a black horizontal line that goes through the configuration with no sample overlap (None).

Through simulations, we showed that PAT was particularly powered to discover variants with a genetic effect in height (Table 1 in the main text). It was hypothesized based on Fig 2 in the main text, that this was because height has a low environmental correlation with the other three traits which were correlated with each other. Here, we explored how environmental correlation impacted discovery power by simulating nine configurations of environmental correlation between the four traits. For each configuration, we simulated one million z-scores and modeled a genetic effect in all four traits. We simulated the effect sizes such that PAT had a power around 30% when there was environmental correlation between all traits. For each of the remaining configurations, we set the sample overlap for a subset of traits to zero (i.e. no environmental correlation).

The resulting simulations are shown in Fig A. The x-axis contains the configurations of environmental correlation where for example $\mathcal{B}, \mathcal{D}, \mathcal{H}, \mathcal{S}$ indicates there was sample overlap in all four traits while $\mathcal{B}, \mathcal{D}$ & $\mathcal{H}, \mathcal{S}$ indicates body mass index and diastolic blood pressure had sample overlap and height and systolic blood pressure had sample overlap but these two sets of traits did not share samples with each other (e.g. BMI and height have no sample overlap). The final value along the x-axis is "None" which indicates no sample overlap between traits which is equivalent to no environmental correlation. The y-axis represents the true positive rate, and each of three methods were

shown in a different color: PAT in blue, MTAG in green, and HIPO in red. All three methods were informed of the genetic and environmental correlation structure. Finally, we included a horizontal black line for each method that goes through the true positive rate for no sample overlap (None).

In Fig A, we saw that across simulations, PAT was the most powerful method. We also observed a jump in power when there was no sample overlap between diastolic blood pressure and systolic blood pressure which have the highest environmental correlation (Table D). While all three methods have a similar oscillation based on which traits have no environmental correlation, HIPO does not seem to respond as strongly as PAT and MTAG. Interestingly, when there was environmental correlation between $\mathcal{B}, \mathcal{H}, \mathcal{S}$ but not $\mathcal{D}$, there was similar power to when there there was no sample overlap (None). While the increase in power was expected, the increase was higher than when there was environmental correlation between $\mathcal{B}, \mathcal{D}, \mathcal{H}$ and no environmental correlation with $\mathcal{S}$. Both of these scenarios only had one trait with no environmental correlation and removed the strongest source of environmental correlation between $\mathcal{D}$ and $\mathcal{S}$. The reason for this larger increase when there was no sample overlap with $\mathcal{D}$ and not with $\mathcal{S}$ was because the second largest source of environmental correlation is between $\mathcal{D}$ and $\mathcal{B}$.

Assessing how the direction of environmental correlation impacts PAT's power

Environmental Correlation	Method		
	PAT	MTAG	HIPO
-50%	22.5%	**23.8%**	23.4%
-25%	17.3%	**17.4%**	17.3%
-10%	**17.5%**	15.8%	15.8%
0%	**17.9%**	15.5%	15.5%
10%	**17.4%**	15.8%	15.8%
25%	17.3%	**17.4%**	17.3%
50%	22.4%	**23.7%**	23.3%

Table E. Comparison of three multi-trait GWAS methods. We simulated seven configurations of one million variants with varying environmental correlation and no genetic correlation between traits. The first column contains the environmental correlation while the remaining three columns report the statistical power of the three methods: PAT, MTAG, and HIPO respectively.

Through simulations, we showed that MTAG and HIPO were better powered to discover variants associated with diastolic and systolic blood pressure, and we hypothesized this was due to the strong environmental correlation between the two traits as it reflected the scenario in Fig 2 in the main text where PAT was conservative in the direction of positive correlation. We were able to further support this in additional simulations shown in Fig A by setting the environmental correlation

between sets of traits to zero. In those simulations, we observed PAT had increased statistical power when there was no environmental correlation between diastolic and systolic blood pressure. In this section, we further explore the impact of environmental correlation by testing whether the correlation direction impacted statistical power. We compared PAT to MTAG and HIPO.

In Table E, we simulated seven configurations of environmental effect between two traits with 1 million simulations in each scenario. All simulation frameworks modeled a genetic effect in both traits and a genetic variance $\sigma_g^2 = 0.25$ for each trait but no genetic correlation. The sample size for each trait was $n = 350,000$ individuals with complete sample overlap. All three methods were informed of the assumed genetic and environmental correlation structure. Here, we observed as environmental correlation increased to 50% as well as when it decreased negatively towards -50% MTAG and HIPO were better powered than PAT to discover associated variants. When there was moderate environmental correlation of 25% as well -25% PAT, MTAG, and HIPO were approximately equally powered. As environmental correlation decreased to 10% and -10% as well as when there was 0% environmental correlation, we found that PAT was more powerful than MTAG and HIPO. We note that while the previous section showed that less environmental correlation reduced statistical power, this section indicated that more environmental correlation resulted in an increase in power for all three methods. The simulations here, however, do not have genetic correlation between the traits which dampens statistical power as shown in Table H.

Empirical M-value Threshold

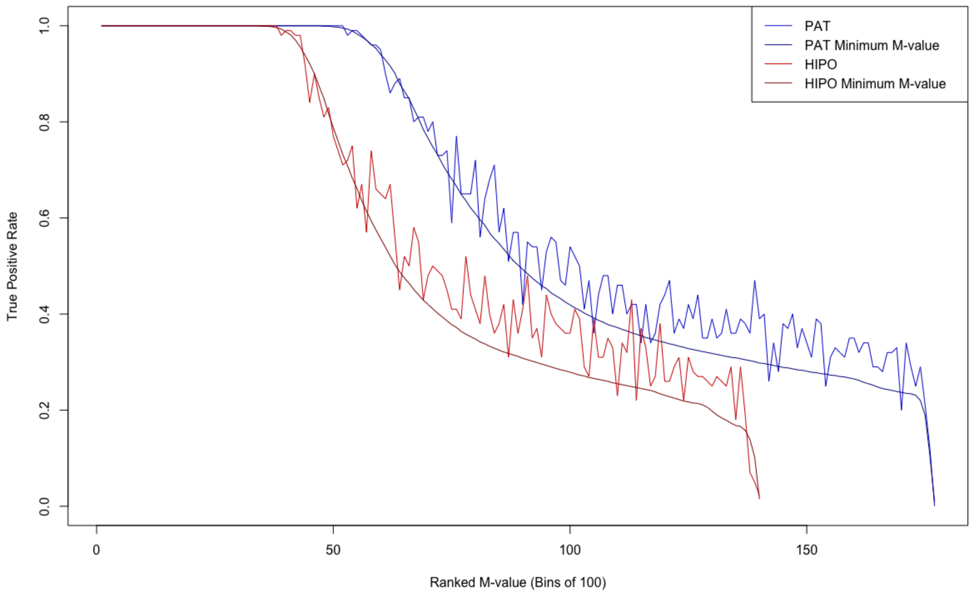

Fig B. Empirical M-value Threshold. The m-value framework was applied to variants identified as associated using PAT and HIPO. The resulting m-values were ranked and binned in sets of 100. For each bin, we plot the smallest m-value as well as the true positive assignment rate for both methods.

Through simulations, we showed that the m-value framework has a low false positive assignment rate. M-values, however, cannot be calibrated the same way p-values can be by using significance

thresholds. Simulations both in the original m-value paper [6] and here support the use of 0.9 as the threshold for interpretation. This is to say, if the m-value is greater than 0.9, the user should interpret the variant as associated with the trait.

While a reasonable threshold, the user would still need to prioritize variants for further exploration and follow up. Here, we explored the true positive rate of the m-value interpretation in ranked order. In the main paper, we presented two tables in the main text (Tables 1 and 2) which contain 1.5 million simulated variants and their z-scores for four traits. 150,000 (10%) of the variants had a genetic effect in at least one trait which were evenly split across all configurations of genetic effect.

In Table 1 in the main text, we recorded that PAT identified 4,405 associated variants while HIPO identified 3,486 associated variants. For both methods, the associated variants were assigned an m-value for each trait; therefore, the respective total number of per trait m-values across four traits was 17,620 and 13,944. For each per trait interpretation, the corresponding ground truth was known; therefore, if the variant genetically affected a trait and the m-value was greater than 0.9, we considered this a true positive. The m-values for each method were ranked from largest to smallest $[1.0, 0.0]$ and binned in sets of 100. For each bin, we calculated the true positive rate and show the results in Fig B.

We observe the true positive rate and the minimum m-value for each bin followed a similar pattern. This means that for a set of per trait associations, the proportion of true positives could be coarsely estimated by the set's minimum m-value. While this approach does not provide the false positive rate for any particular m-value, it may provide some guidance on whether a particular subset of variants has a high enough true positive rate to warrant further analysis.

To make this explanation more concrete, the true positive rate for PAT in the first 52 bins (5,200 m-values) was 100% for each. Using three digits of precision, the first 47 bins have a minimum m-value of 1.000. While bins 48-51 also have a true positive rate of 100%, the minimum m-value observed for those bins was less than 1.000 with the minimum m-value for bin 52 being 0.995. While the ground truth for the 52nd bin was zero false positive, we could have used the minimum m-value to estimate that approximately one of the m-values was a false positive. This approach could be used as a rough lower bound when determining whether further exploration of a particular set of variants is warranted. From this, a rough estimate for all m-values greater than 0.9 is a 90% true positive rate. This is far more conservative than necessary as we empirically showed a total false positive rate of 0.92% or less (Table 2 in the main text). Instead, by using smaller bins (such of size 100) the user can have a crude estimate of the true positive rate of that bin of m-values.

Ranked false positive rate between m-values and p-values

In the main paper, we compared the per trait m-values produced for PAT and HIPO to the per trait p-values produced by MTAG. In Table 2 in the main text, PAT identified 6,264 true per trait associations. Applying m-values resulted in 4,557 true per trait associations for HIPO while MTAG discovered 3,064. While these results indicated PAT was the most powerful approach, they also provided evidence that computing posterior predictions (m-values) after omnibus associations was a more powerful approach to association testing than directly analyzing each trait using MTAG.

While the number of true positives supported this claim, the difference in the false positive rate between m-values and p-values drew this claim into question. This is to say, if the same number of false positives were produced by m-values and MTAG's p-values, it is possible that MTAG would be the most powerful approach.

In order to test this claim, m-values must be modified to better reflect p-values. Currently,

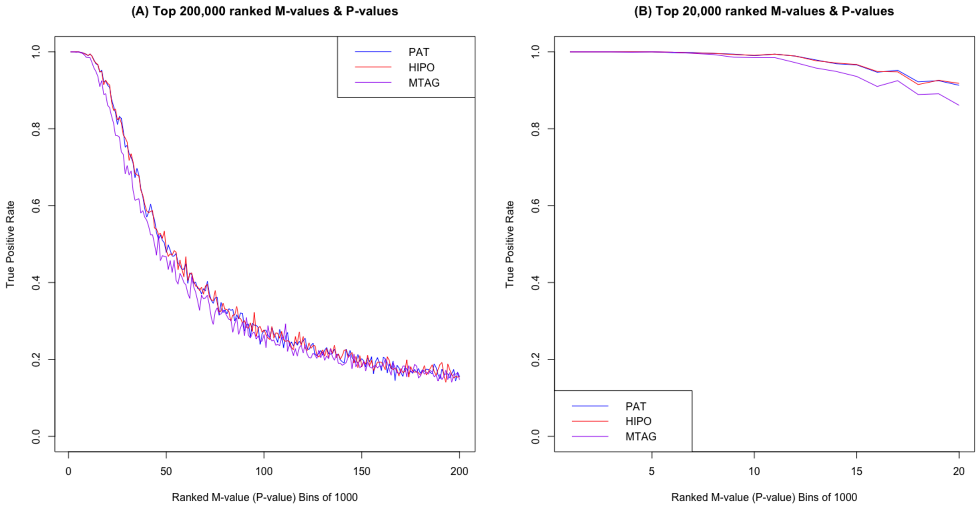

Fig C. Comparison of m-values and p-values. M-values were assigned to all z-scores for PAT and HIPO. For each method, they were ranked and placed in bins of 1,000. The p-values from MTAG were also ranked and binned in sets of 1,000. A comparison of their respective true positives rates are shown in the first 200 bins and first 20 bins, A and B respectively.

m-values were only interpreted for variants deemed genome-wide significant. This was due to their design as an interpretation framework. M-values were not designed to replace p-values but to elucidate what traits may be driving the significant association. In this comparison, we assigned m-values for every p-value instead of only to the omnibus significant ones. This enabled a comparison of all posterior predictions to the per trait p-values of MTAG. While illustrative of the power of m-values, this approach is not advisable in practice. M-values should only be used as a means of interpreting omnibus associations.

For this comparison, we used the 1.5 million simulations with 10% causal variants previously described in the main results. The 150,000 causal variants were equally split across all configurations of genetic effect. For each of the configurations, three different effect sizes were modeled. As stated previously, MTAG directly produced a per trait p-value and m-values were assigned to HIPO and PAT.

In Fig C, we ranked the m-values for PAT and HIPO and the p-values for MTAG. For p-values, the rank order was from $[0.0, 1.0]$ while m-values were ordered from $[1.0, 0.0]$. For each m-value (and p-value), whether or not the variant was truly associated with the trait was known; therefore, the true positive rate for the variants in rank order could be calculated. After ranking the m-values (and p-values), the variants were binned in sets of 1,000 for each method. In Fig CA, the first 200 bins or 200,000 top p-values (m-values) for each method are along the x-axis. Along the y-axis, the true positive rate for each bin is shown. For the most significant associations by p-values and m-values the true positive rate was 1.00. As the ranked position decreased, the true positive rate decreased. Overall, the general trend of m-values for both HIPO and PAT and p-values for MTAG followed the same pattern. In the top left of Fig CA MTAG (purple) has a slightly lower true positive rate than PAT (blue) and HIPO (red) which overlay each other.

In Fig CB, we explored the top 20 bins or 20,000 m-values and p-values more closely. Here, we saw that there was some separation between the m-values for PAT and HIPO, respectively shown in blue and red, and MTAG in purple. From this, there is evidence that while the m-value framework did not control for false positives directly, it does have increased power relative to a

directly interpretable multi-trait method, such as MTAG. We note that while true, neither the true (nor false) positive rate can be elucidated from the m-value directly. Therefore, m-values should only be used as designed to interpret significant p-values.

Omnibus Associations in the UK Biobank

Set of Traits	Single Trait GWAS	HIPO	MTAG	MI GWAS	PAT
$\mathcal{B}, \mathcal{D}, \mathcal{H}, \mathcal{S}$	176	170	**176**	**176**	**176**
$\mathcal{B}, \mathcal{D}, \mathcal{H}$	165	161	**165**	**165**	**165**
$\mathcal{B}, \mathcal{D}, \mathcal{S}$	916	709	**916**	914	908
$\mathcal{B}, \mathcal{H}, \mathcal{S}$	107	105	**107**	105	**107**
$\mathcal{D}, \mathcal{H}, \mathcal{S}$	1,690	1,682	1,676	1,689	**1,690**
$\mathcal{B}, \mathcal{D}$	1,388	1,059	**1,388**	1,221	1,275
$\mathcal{B}, \mathcal{H}$	4,911	4,974	4,857	4,851	**4,911**
$\mathcal{B}, \mathcal{S}$	575	494	**560**	545	542
$\mathcal{D}, \mathcal{H}$	2,434	2,426	2,420	2,430	**2,434**
$\mathcal{D}, \mathcal{S}$	4,921	4,785	4,653	**4,855**	2,519
$\mathcal{H}, \mathcal{S}$	1,481	1,377	1,471	1,448	**1,481**
$\mathcal{B}$	28,967	22,534	**24,741**	22,525	10,713
$\mathcal{D}$	6,903	4,951	**5,473**	5,367	2,227
$\mathcal{H}$	149,263	109,066	108,681	131,761	**147,146**
$\mathcal{S}$	7,649	3,377	**6,048**	5,617	1,723
None	0	19,829	931	0	**22,095**
Total	211,546	177,519	164,263	183,669	**200,112**

Table F. UK Biobank data analysis. The first column lists the set of traits. Each remaining column corresponds to different GWAS methods with the second column showing the Single Trait GWAS results. These results were generated from individual level data and produced the summary statistics. The remaining columns show how many of these variants were also identified by HIPO, MTAG, MI GWAS, and PAT, respectively. We separate the data according to Single Trait GWAS significant results; for example, the first row of results is the number of variants found associated with all traits $\mathcal{B}, \mathcal{D}, \mathcal{H}, \mathcal{S}$ (body mass index, diastolic blood pressure, height, and systolic blood pressure).

In this real data analysis, we analyzed summary statistics for body mass index ($\mathcal{B}$), diastolic blood pressure ($\mathcal{D}$), height ($\mathcal{H}$), and systolic blood pressure ($\mathcal{S}$) measured in the UK Biobank. Here, five methods were compared: Single Trait GWAS (how the z-scores and p-values were derived), HIPO, MTAG, MI GWAS, and PAT [7, 8]. There were 7,025,734 variants which were biallelic, have

non-ambiguous strands, a minor allele frequency greater than 1%, and an INFO score greater than 80%. The reference and alternate allele were coordinated across traits by flipping the direction of the effect when necessary. LD-Score regression and cross-trait LD-Score regression were used to calculate the genetic and environmental covariance structure (see above) [4, 5].

The first column lists all subsets of the four traits while the second column contains the results from Single Trait GWAS. This is the maximum number of variants the other four methods could recapture. Each row was based on which traits were identified as associated using individual level data for each trait separately. For example, the first row shows the results for body mass index ($\mathcal{B}$), diastolic blood pressure ($\mathcal{D}$), height ($\mathcal{H}$), and systolic blood pressure ($\mathcal{S}$). There were 176 unique variants found to be significantly associated in all four traits by Single Trait GWAS; therefore, HIPO MTAG, MI GWAS, and PAT could not have a value greater than 176 in the first row. In Table F, we see every method except HIPO was able to identify all 176 variants as associated with at least one of the four traits. We note that while MTAG is not an omnibus method, a variant was deemed associated as long as one trait is significantly associated. Additionally, HIPO computes four components for testing. If at least one component was genome-wide significant, the variant was interpreted as associated. All methods were tested at $\alpha = 5 \times 10^{-8}$ and bound by the original single trait results to provide a fair comparison to fundamentally different methods.

When we considered the sets of traits identified by Single Trait GWAS, we see general consistency with simulations shown in Table 1 in the main text. PAT was well powered when there was an effect in height. We see general consistency between MTAG and HIPO with MTAG identifying more variants than HIPO. Finally, when we considered variants not originally found by the Single Trait GWAS. Here, MI GWAS found no new associations, but this was by design. MTAG, however, was able to identify 931 additional variants. HIPO identified 19,829 while PAT discovered 22,095.

Overall, 37,890 novel associations were discovered by three of the multi-trait methods (HIPO, MTAG, and PAT). 385 of these variants were found by all three methods. 17,788 out of the 22,095 variants discovered by PAT were unique to PAT. For HIPO, 15,407 out of its 19,829 variants were unique while 115 of MTAG's 931 associations were only identified by MTAG. While PAT was the most powerful in regards to identifying novel associations, these results indicated HIPO, MTAG, and PAT were able to identify many unique variants due to their differing model designs.

We note that these results were presented as the number of unique variants instead of independent loci implicated. This was done to better understand which set of variants each method identified as shown in Table F. (Otherwise, all variants in the locus would need to be truly associated with the same set of traits.) With that in mind, we report the number of unique loci identified genome-wide across traits. Single Trait GWAS returned the most independent loci (1,512) while the multi-trait methods, HIPO, MTAG, MI GWAS and PAT identified 1,340, 1,262, 1,347, and 1,324 respectively.

Computational speedup with importance sampling

We now show how the cost of null simulations can be reduced using importance sampling. When setting the critical value κ for PAT's likelihood ratio test, the data is simulated according to the null distribution $\mathcal{N}(0, \Sigma_e)$. As a result, a likelihood ratio greater than κ is expected only $\alpha \times n$ times. As GWAS uses the significance threshold of $\alpha = 5 \times 10^{-8}$, the number n needs to extremely large to ensure replication of results, in practice $n = 10^{10}$. Simulating and storing 10^{10} vectors of summary statistics is computationally expensive, especially in terms of memory. This burden can be reduce using importance sampling where the null data is simulated according to a different distribution $\mathcal{N}(0, r\Sigma_e)$ where r is a scaling factor that increases the number of samples that are

Set of Traits (Variance)	Number of Simulations	Scaling of Covariance Matrix ($r \times \Sigma_e$) 5	6	7	8	9	10
$\mathcal{B},\mathcal{D},\mathcal{H},\mathcal{S}$ (9e-07)	1e6	6.94	13.82	6.88	12.10	6.49	6.12
	1e5	0.741	1.01	1.40	**1.01**	0.58	0.70
	1e4	0.046	0.12	0.13	0.06	0.09	0.10
$\mathcal{B},\mathcal{D},\mathcal{H}$ (5e-07)	1e6	5.27	4.92	7.50	7.11	13.76	7.22
	1e5	0.54	0.53	0.58	**1.15**	1.00	0.69
	1e4	0.035	0.045	0.045	0.074	0.096	0.08
$\mathcal{B},\mathcal{D},\mathcal{S}$ (2e-07)	1e6	11.11	7.93	9.35	8.32	11.10	12.07
	1e5	0.62	0.81	1.14	**1.17**	0.90	0.94
	1e4	0.10	0.08	0.16	0.13	0.17	0.18
$\mathcal{B},\mathcal{H},\mathcal{S}$ (4e-07)	1e6	3.20	11.75	5.53	6.22	9.29	6.08
	1e5	0.33	0.65	0.64	**0.93**	0.63	0.73
	1e4	0.07	0.06	0.09	0.07	0.09	0.22
$\mathcal{D},\mathcal{H},\mathcal{S}$ (9e-07)	1e6	7.23	7.97	18.51	18.31	16.39	12.73
	1e5	0.54	0.73	0.92	**1.30**	1.26	1.47
	1e4	0.10	0.08	0.11	0.08	0.09	0.31
$\mathcal{B},\mathcal{H}$ (6e-07)	1e6	10.67	12.22	22.48	22.551	20.586	18.295
	1e5	0.59	0.83	0.88	**1.72**	3.02	2.04
	1e4	0.10	0.13	0.20	0.16	0.22	0.27
$\mathcal{D},\mathcal{S}$ (6e-08)	1e6	28.83	21.53	110.27	27.46	33.39	72.58
	1e5	1.492	4.04	3.49	**4.58**	7.39	5.05
	1e4	0.18	0.37	0.40	0.63	0.51	0.67

Table G. Stable estimates of critical values in fewer null simulations. We generate the critical value κ at $\alpha = 5 \times 10^{-8}$ 25 times for various combinations of four traits: body mass index ($\mathcal{B}$), diastolic blood pressure ($\mathcal{D}$), height ($\mathcal{H}$), and systolic blood pressure ($\mathcal{S}$). We simulated data according to $\mathcal{N}(0, r\Sigma_e)$ for $r = \{5, 6, 7, 8\}$ and for $n = 10^4$, 10^5 and 10^6 simulations. We then take a ratio of the variation in the estimated critical value κ which we call the stability. The first column is the set of traits and the variance for $\mathcal{N}(0, 1\Sigma_e)$ using $n = 10^{10}$ simulations. The second column is the number of simulations while the remaining columns show the stability for different scaling factors of the covariance matrix $r : r = \{5, 6, 7, 8\}$.

significant. We note that importance sampling adjusts the weights of the samples in estimating the p-values (see Methods). If r is well chosen κ can be set with fewer simulations. In Table G, the critical value κ was estimated 25 times and the sample variance of these estimates provided a measure of the stability of the sampling. This was repeated for different values of the scaling factor r and number of samples n. We defined the ratio of the sample variance using importance sampling to the sample variance of null simulations as stability. When the ratio was close to one, the estimated κ using importance sampling was as stable as the κ estimated directly using null simulations and values larger than one indicated importance sampling had a smaller variance. Four traits: body mass index ($\mathcal{B}$), diastolic blood pressure ($\mathcal{D}$), height ($\mathcal{H}$), and systolic blood

pressure ($\mathcal{S}$) were considered in these simulations as well as subsets of the traits. When using 10^6 simulations, we found using importance sampling was consistently more stable for all reported scaling factors, r. For diastolic blood pressure and systolic blood pressure, importance sampling was also more stable for all reported scaling factors using 10^5 simulations. When using the scaling factor $r = 8$ for $n = 10^5$ simulations, the variance for the value κ when using importance sampling was approximately equal to the variance using 10^{10} null simulations across the various sets of traits. For most sets of traits importance sampling was still slightly more stable; it was only for body mass index, height, and systolic blood pressure that it was less stable with a ratio of 0.93 which is still very close to 1. This means, the same stability could be achieved using only 10^5 simulations which is 10^5 fewer simulations. This reduction in computational resources holds true across data sets. In practice, however, the use of 10^6 simulations is more practical as the stability of the critical value κ is less sensitive to the setting of r. This still results in using $10,000$ fewer simulations.

Further intuition on PAT's rejection region with comparisons to three methods

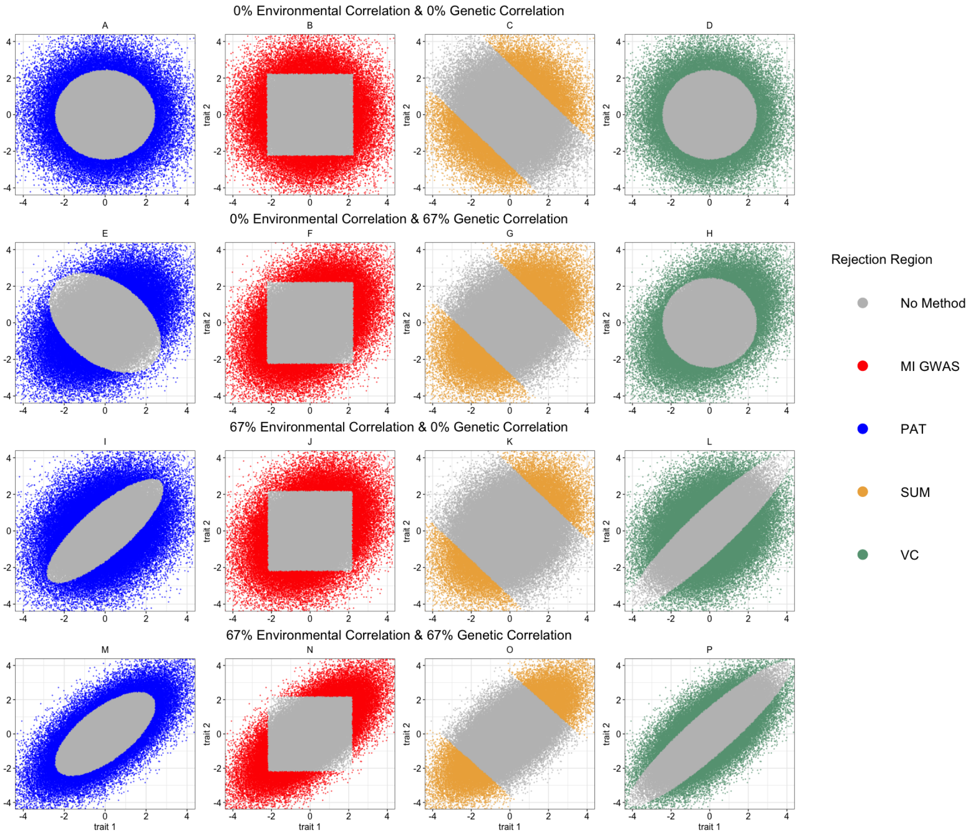

Fig D. Four multi-trait methods' rejection regions across four simulation frameworks. We simulated 100,000 summary statistics for two traits and set the genetic variance equal. We varied the genetic and environmental correlation. Each row corresponds to a different set of simulations while each column and color highlights a different method.

In the main paper, we compared PAT and MI GWAS by simulating 100,000 summary statistics across four configurations of genetic and environmental correlation for two traits (see Fig 2 in

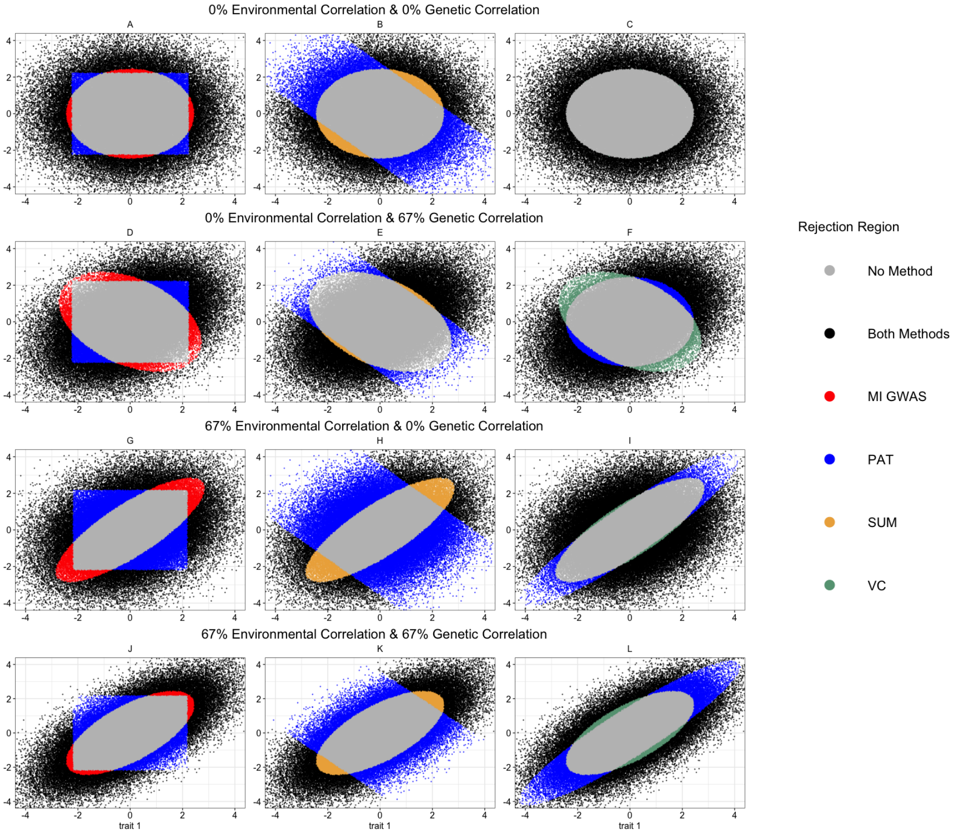

Fig E. Comparison of the rejection region between PAT and three multi-trait methods across four simulation frameworks. We simulated 100,000 summary statistics for two traits and set the genetic variance equal. We varied the genetic and environmental correlation. Each row corresponds to a different simulation framework while each column is a comparison between PAT and one other method. Across all configurations, variants identified by both PAT and the other method are shown in black, those both methods missed are grey, and the variants unique to PAT are in blue. In the first column, variants unique to MI GWAS are red, in the second column variants unique to SUM are yellow, and in the third column variants unique to VC are in green.

the main text). Here, we present these same simulations and included two additional methods: SUM and VC [9]. SUM is a method designed to identify homogeneous effects amongst traits and computes a weighted sum of z-scores using the between-trait correlation as the weight. The other method, VC (variance component), is better suited for discovering heterogeneous effects. Instead of modeling a fixed effect, the method assumes the effect in each trait is drawn centered around the overall mean effect with the covariance defined by the between-trait covariance.

In Fig D, each row corresponds to a different simulation framework and each method has its own column. The first row (Fig DA-D), contains the simulations with 0% environmental correlation and 0% genetic correlation while the second row (Fig DE-H) is when there is 0% environmental correlation and 67% genetic correlation. The third row (Fig DI-L) contains the simulations with 67% environmental correlation and 0% genetic correlation while the fourth row (Fig DM-P) has the simulations with 67% environmental correlation and 67% genetic correlation. For each simulation framework, we showed the rejection region of four multi-trait methods with each method in its own column and variants missed by the method in grey. In the first column, we present PAT and color

Method	Genetic Correlation	0% Environmental Correlation			67% Environmental Correlation		
		MI GWAS	SUM	VC	MI GWAS	SUM	VC
No Method	0%	71,840	69,682	74,119	69,715	71,335	70,461
Both Methods		22,543	14,422	25,854	20,477	24,346	22,557
PAT		3,311	11,432	0	6,468	2,599	4,388
Other Method		2,306	4,464	27	3,340	1,720	2,594
No Method	67%	56,933	56,492	59,642	70,917	69,673	69,994
Both Methods		21,077	8,662	35,603	19,504	14,395	16,414
PAT		17,462	29,877	2,936	6,350	11,459	9,440
Other Method		4,528	4,969	1,819	3,229	4,473	4,152

Table H. Numerical values of the comparison of the rejection region between PAT and three multi-trait methods. This table is the numerical values that correspond to Fig E. The first column indicates which method was able to reject the null. Both Methods, No Method, PAT, or the "Other Method". The second column is the genetic correlation while the remaining columns correspond to a different multi-trait method compared to PAT (i.e. the "Other Method"). Columns 3-5 and 6-8 are split by environmental correlation.

variants discovered by the method as blue while in the second column, we present MI GWAS and color code the variants found as red. In the third column variants discovered by SUM are yellow, and the fourth column is VC and has the variants it discovered in green.

Here, we observed that the variance component methods: PAT and VC were elliptical in shape. MI GWAS as previously described (see Fig 2 in the main text) was a square due to rejecting the null based on the trait with the minimum p-value while SUM was well powered in the direction of positive correlation and powerless when the correlation between traits was negative (e.g. x=-1, y=1). PAT was the only method whose rejection region changed with each adjustment to the genetic and environmental correlation. The other three methods only required knowledge of the null distribution (i.e. environmental correlation) to set their rejection regions; therefore, rows one and two have identical rejection region shapes and rows three and four have identical shapes for all methods except PAT.

We further explored these simulations by comparing the three methods: MI GWAS, SUM, and VC to PAT and present these results in Fig E with the numerical values in Table H. Each row corresponds to one simulation framework and each column is a comparison between PAT and one other method. Variants discovered by both methods are shown in black, those missed by both methods are grey, and variants uniquely discovered by PAT are blue. In column one, we show a comparison between PAT and MI GWAS and is identical to the third column of Fig 2 in the main text; variants discovered by MI GWAS are red. The second column compares PAT and SUM with the variants unique to SUM are yellow. The third column compares PAT and VC and has the variants unique to VC in green.

In Fig E, we saw that different rejection regions were better equipped to discover different variants. The first column was extensively discussed in the main paper, so here we focus on the second and third column. When we considered the comparison of PAT and SUM in the second column, we saw that PAT was more powerful when the summary statistics were negatively correlated; in this direction, SUM was powerless. When the summary statistics were positively correlated, SUM consistently discovered variants PAT failed to identify across simulation frameworks. Finally, in the third column we compared PAT and VC. These two methods were elliptical in shape and approxi-

mately identical in Fig EC. In Fig EF, PAT had more power in the direction of genetic correlation while VC had more power when summary statistics were negatively correlated which was similar to MI GWAS (Fig ED). However, when there was environmental correlation, as shown in rows three and four, MI GWAS was more powerful in the direction of environmental correlation and PAT was more powerful in the direction of negative correlation (Fig EG and Fig EJ). This was flipped from what was observed in Fig ED. For VC, there was no flip in the direction of power. PAT was more powerful than VC in the direction of environmental correlation while VC was more powerful in the direction of negative correlation. While PAT and VC account for the environmental correlation in a similar manner, PAT also considered the genetic correlation which further shapes the rejection region which resulted in differing power between the methods.

PAT controls false positives

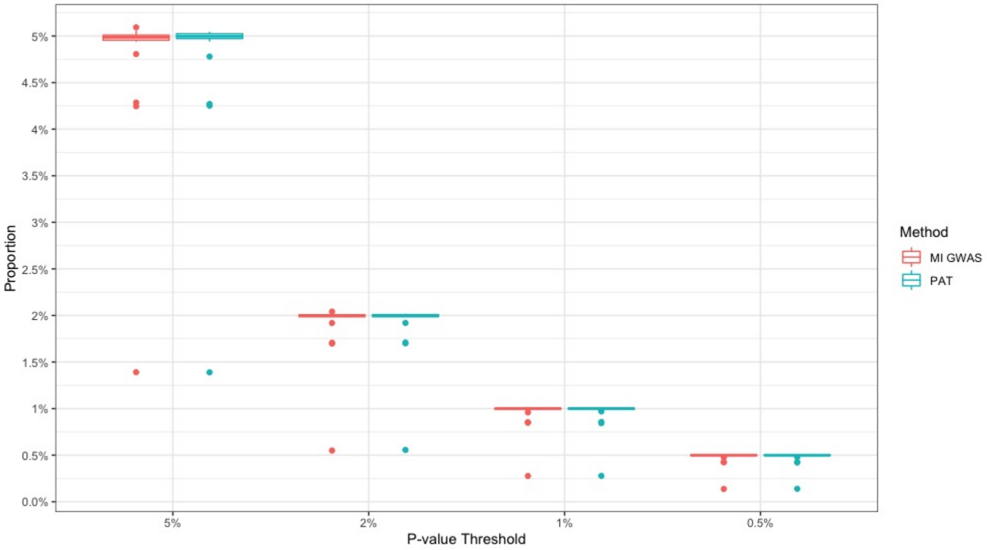

Fig F. Comparison of false positive rate for PAT and MI GWAS. We use the UK Biobank data to estimate the genetic and environmental covariance matrices for four traits (body mass index, diastolic blood pressure, height, and systolic blood pressure) and inform PAT of the covariance structure between traits. We then simulate 10^8 summary statistics 25 times with only environmental covariation between the traits. We examine the number of variants with p-values below 5%, 2%, 1%, and 0.5% level of significance to test how effectively the methods control false positives.

In the main paper, we provided some intuition on PAT as well as compared it to two other multi-trait methods: HIPO and MTAG. Here and in the following sections, we provided further simulations and compared PAT to MI GWAS (multiple independent GWAS) in order to further explore the performance of PAT. We selected four quantitative traits: body mass index ($\mathcal{B}$), diastolic blood pressure ($\mathcal{D}$), height ($\mathcal{H}$), and systolic blood pressure ($\mathcal{S}$) from the UK Biobank to simulated z-scores reflective of real data (Tables A and B) [1]. We simulated 10^8 summary statistics with 25 replications according to the environmental correlation estimated from the UK Biobank. While the simulations only have environmental correlation, PAT also models the alternative hypothesis which assumed a genetic effect and genetic correlation between the traits. Here, the calibration of MI GWAS was also checked, but this method made no assumptions about the phenotypic covariance between traits. Fig F shows box-plots of the proportion of p-values below a significance threshold

α for PAT and MI GWAS. The threshold α was set to the following values: 5%, 2%, 1%, and 0.5%. As the distribution of p-values under the null hypothesis is uniform, 5% of p-values were expected to be smaller than the level of significance, $\alpha = 5\%$. This expectation holds for all levels of significance under the null. In Fig F, both methods were shown to behave within expectation at the various levels of significance. This indicates both methods are effective at controlling the false positive rate.

PAT increases power for pleiotropic effects

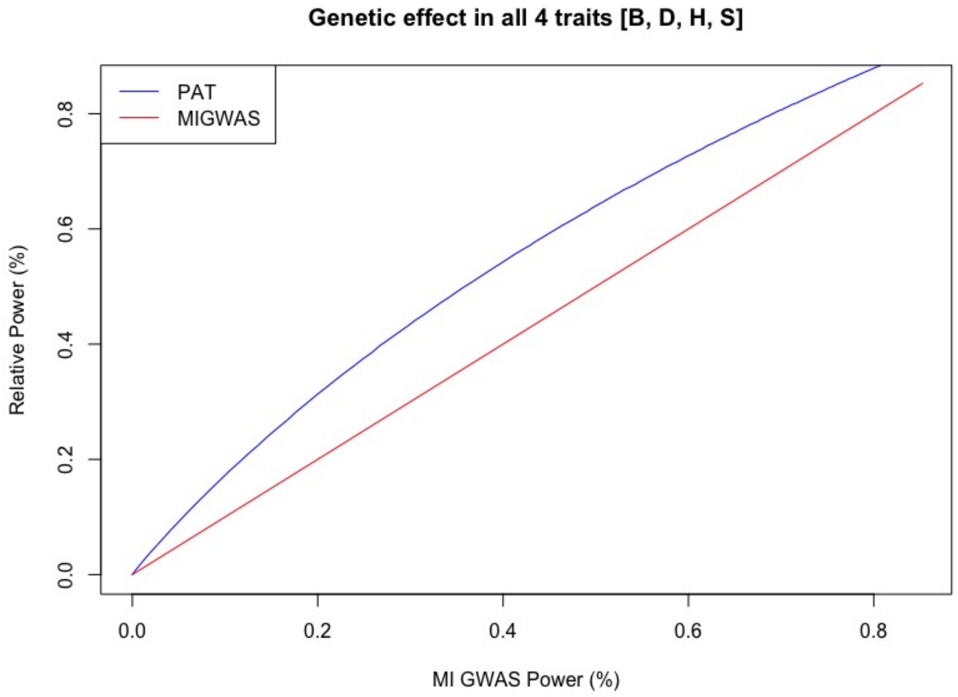

Fig G. Comparison of relative power between PAT and MI GWAS when there is a genetic effect in every trait. We use the UK Biobank to estimate the genetic and environmental covariance matrices of four traits. The simulated effect sizes begin by following the polygenic model and then increase by a magnitude of 25 until the effect size is 5,000 times larger than the polygenic model. The power of PAT and MI GWAS to identify associated variants are shown relative to the power of MI GWAS.

PAT is a likelihood ratio test whose rejection region is elliptical while the rejection region for MI GWAS is a square as shown in Fig 2 in the main text. Here, instead of comparing the shape, we use simulations to understand the relative power of each method when analyzing simulations based on four UK Biobank traits. While PAT is able to account for overlapping sample sizes (see Methods), in these simulations individuals are assumed to be uniquely measured for each trait. This means there is no environmental correlation either in the simulations or when setting the test statistics for PAT (and MI GWAS). We make this assumption due to MI GWAS being unable to account for overlapping samples and to enable a fairer comparison of the methods.

To compare the power of MI GWAS and PAT, we simulate 10^8 z-scores as if there is a genetic effect in every trait. The simulated genetic effect sizes begin by following the polygenic model and

then for each subsequent set of 10^8 z-scores the genetic effect sizes increases by a magnitude of 25. This is repeated 200 times until it is 5,000 times larger than the polygenic model. In Fig G, the power of MI GWAS and PAT are shown relative to the power of MI GWAS. When variants genetically affect all traits, PAT has more power than MI GWAS regardless of the genetic effect size. Furthermore, while power increases for both methods as effect sizes increase, PAT has a faster increase in power relative to MI GWAS. These results indicate that when pleiotropy is present, PAT has more power than MI GWAS to detect variants with weaker effect sizes.

PAT outperforms MI GWAS for many misspecified models of genetic effect

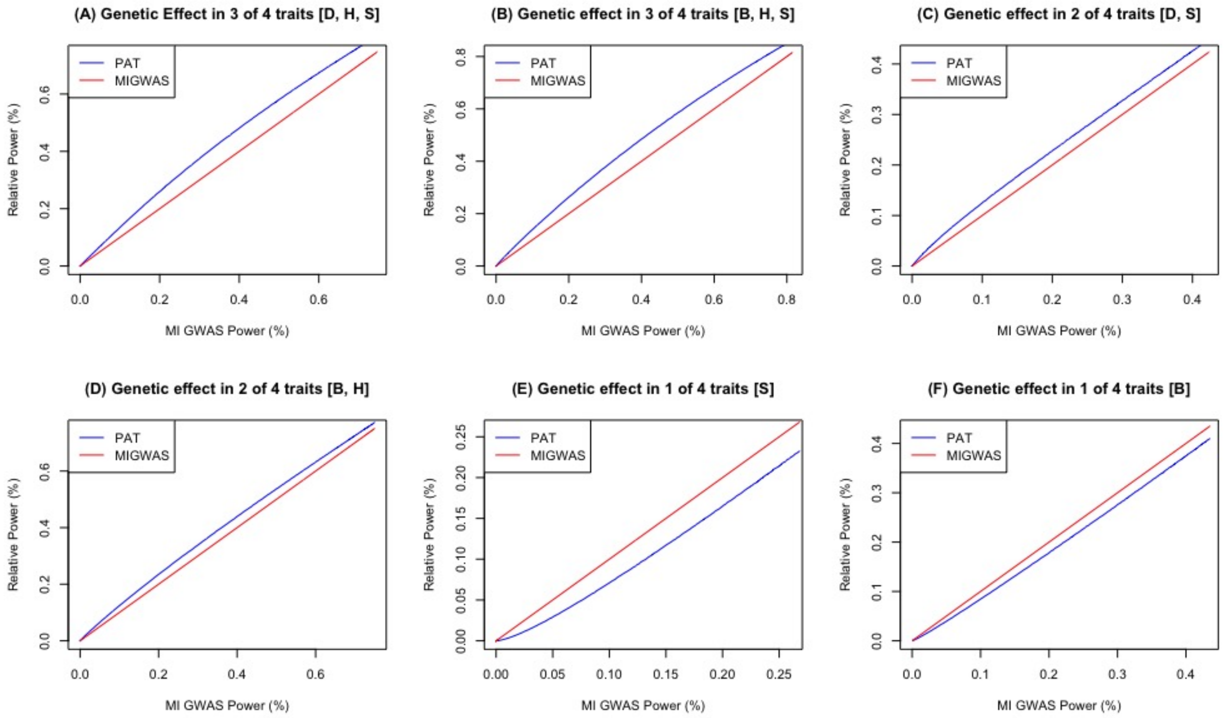

Fig H. Comparison of statistical power when only a subset of traits have a genetic effect. These simulations are based off for four UK Biobank traits: body mass index (B), diastolic blood pressure (D), height (H), and systolic blood pressure (S). Each sub-figure show different sets of traits having their genetic effect set to zero. The relative performance of PAT is compared to MI GWAS with the power of MI GWAS as the baseline. A and B depict simulations which model a genetic effect in three of the four traits while C and D show a genetic effect occurs in only half of the traits. In the last, E and F, there is a genetic effect in only one of the traits.

While PAT has more statistical power when the true underlying distribution of genetic effects is known, it is unreasonable to assume every associated variant affects all traits. We, therefore, test the robustness of PAT to model misspecificiaton. PAT is informed of two models, the null model (no genetic effect) and the full model (genetic effect in all traits). The simulations are based on four UK Biobank traits but assume no environmental correlation. The z-scores are then simulated

to violate the assumed alternative model by having a genetic effect in only a subset of the traits. The results can be seen in Fig H where the relative power of PAT is compared to MI GWAS under various effect sizes.

Fig H explores the relative power for PAT and MI GWAS for six different configurations of effect. Sub-figures A and B model the case when there is a genetic effect in three of the four traits. For Fig HA, the genetic effect size for body mass index is set to zero and for Fig HB there is no genetic effect in diastolic blood pressure. Under both of these conditions, PAT has a substantial improvement in power over MI GWAS. For Fig HC and Fig HD the simulations model a genetic effect in only two of the traits. In Fig HC, the genetic effect size for body mass index and height are set to zero while in Fig HD, there is no genetic effect for diastolic and systolic blood pressure. PAT is still more powerful than MI GWAS though the advantage is more modest. In the remaining two, Fig HE and Fig HF, there is a genetic effect in only one trait. In Fig HE, there is a genetic effect in systolic blood pressure while Fig HF models there being an effect in only body mass index. When there is no pleiotropy, PAT is less powerful than MI GWAS. These simulations indicate that when pleiotropy is present, it is advantageous to jointly model genetic effects across traits, but this advantage decreases as the amount of pleiotropy decreases.

References

1. Neale Lab B. UK Biobank Summary Statistics; 2017. http://www.nealelab.is/blog/2017/7/19/rapid-gwas-of-thousANDs-of-phenotypes-for-337000-samples-in-the-uk-biobank.

2. Neale Lab B. UK Biobank Summary Statistics; 2018. http://www.nealelab.is/uk-biobank/.

3. Consortium TGP. A global reference for human genetic variation. Nature. 2015;526:68–74.

4. Bulik-Sullivan BK, Loh PR, Finucane HK, et al. LD Score regression distinguishes confounding from polygenicity in genome-wide association studies. Nature Genetics. 2015;47(3):291–295. doi:10.1038/ng.3211.

5. Bulik-Sullivan B, Finucane HK, Anttila V, et al. An atlas of genetic correlations across human diseases AND traits. Nature Genetics. 2015;47(11):1236–1241. doi:10.1038/ng.3406.

6. Han B, Eskin E. Interpreting Meta-Analyses of Genome-Wide Association Studies. PLOS Genetics. 2012;8(3):1–11. doi:10.1371/journal.pgen.1002555.

7. Qi G, Chatterjee N. Heritability informed power optimization (HIPO) leads to enhanced detection of genetic associations across multiple traits. PLOS Genetics. 2018;14(10):1–21. doi:10.1371/journal.pgen.1007549.

8. Turley P, Walters RK, Maghzian O, et al. Multi-trait analysis of genome-wide association summary statistics using MTAG. Nature Genetics. 2018;50(2):229–237. doi:10.1038/s41588-017-0009-4.

9. Liu Z, Lin X. Multiple phenotype association tests using summary statistics in genome-wide association studies. Biometrics. 2018;74(1):165–175. doi:https://doi.org/10.1111/biom.12735.
