# Supplementary material for: Leveraging pleiotropy for joint analysis of genome-wide association studies with per trait interpretations
Source: PLoS Genet. 2022 Nov 7;18(11):e1010447. doi: 10.1371/journal.pgen.1010447 (PMC9671458; doi:10.1371/journal.pgen.1010447)
Supplement: Attachment — Submitted filename: ReviewerResponse.pdf [file pgen.1010447.s002.pdf]

UNIVERSITY OF CALIFORNIA, LOS ANGELES

BERKLEY • DAVIS • IRVINE • LOS ANGELES • MERCED • SANTA BARBARA
RIVERSIDE • SAN DIEGO • SAN FRANCISCO • SANTA CRUZ

July 13, 2022

Dear Dr. Epstein and Dr. Balding,

Thank you for considering our manuscript, “Leveraging pleiotropy for joint analysis of genome-wide association studies with per trait interpretations”, for publication in PLOS Genetics. We would like to thank the three reviewers for their thoughtful feedback and comments. Following the reviewers’ suggestions, we have made extensive edits to our manuscript primarily in the results and discussion sections. Details are included below with inline responses to the reviewers in red with new text in yellow highlight.

In brief, we incorporated the following new analyses: simulations exploring the impact of environmental correlation on statistical power, the inclusion of the multi-trait method, ASSET in the main simulations, and a comparison of PAT’s rejection regions to two multi-trait methods, VC and SUM. Additionally, we edited the writing to be clearer.

We thank the reviewers for their suggestions, as we believe these changes have substantially improved the manuscript. We would also like to thank the editors again for considering our revised manuscript.

Sincerely,

Eleazar Eskin
Professor
University of California, Los Angeles

Reviewer #1: ## Overview
This work introduces a new likelihood-based approach, PAT, for GWAS summary statistic data to test the global null hypothesis of no association across multiple traits. PAT uses estimates of the genetic covariance from cross-trait LD-score regression to calculate a likelihood ratio test statistic where the p-values are estimated using a computationally efficient approach. The authors find that PAT makes overall more discoveries compared to MTAG/HIPO on simulated and real data. In addition to PAT, the authors calculate posterior probabilities for each trait to help determine which traits may be driving statistical significance by extending a previous quantity called m-value.

We thank the reviewer for their suggestions on writing and further analyses, and we respond point by point below.

## Major comments

- Can the authors make the software/reproducible code for the analysis/simulations available?

We thank the reviewers for pointing out our oversight. We have now included the github link in the paper which includes both the software and simulation frameworks.

PAT is freely available at https://github.com/koditaraszka/pat [pg. 2].

- There should be a set of simulations exploring the impact of the environmental correlation among traits since PAT's improvements seem to stem from low environmental correlation (or is it because height has a negative genetic correlation with the other traits?). In either case, I think this should be explored a bit more in the paper. For example, varying the proportion of traits with no environmental correlation from 0, 1/4, 1/2, 3/4, 1 and the magnitude of the correlation (none, low, moderate, high). I think this is important to include as a motivator for PAT and the prevalence of such analysis in practice. It could also be worth exploring the direction of the genetic correlations as that may be important in the method comparisons.

Thank you for the suggestion to explore the impact of environmental correlation among traits. The reviewer is correct that this ability to properly account for environmental correlation is a major motivator for PAT. We added the following section to the Supplementary Materials:

**Assessing the role of environmental correlation on PAT's power**
Through simulations, we showed that PAT was particularly powered to discover variants with a genetic effect in height (Table 1). It was hypothesized based on Fig 1, that this was because height has a low environmental correlation with the other three traits which were correlated with each other. Here, we explored how environmental correlation impacted discovery power by simulating nine configurations of environmental correlation between the four traits. For each configuration, we simulated one million z-scores and modeled a genetic effect in all four traits. We simulated the effect sizes such that PAT had a power around 30% when there was environmental correlation between all traits. For each of the remaining configurations, we set the sample overlap for a subset of traits to zero (i.e. no environmental correlation).

The resulting simulations are shown in Supplementary Fig 1. The x-axis contains the configurations of environmental correlation where for example B,D,H,S indicates there was sample overlap in all four traits while B,D & H,S indicates body mass index and diastolic blood pressure had sample overlap and height and systolic blood pressure had sample overlap but these two sets of traits did not share samples with each other (e.g. BMI and height have no sample overlap). The final value along the x-axis is "None" which indicates no sample overlap

between traits which is equivalent to no environmental correlation. The y-axis represents the true positive rate, and each of three methods were shown in a different color: PAT in blue, MTAG in green, and HIPO in red. All three methods were informed of the genetic and environmental correlation structure. Finally, we included a horizontal black line for each method that goes through the true positive rate for no sample overlap (None).

In Fig 1, we saw that across simulations, PAT was the most powerful method. We also observed a jump in power when there was no sample overlap between diastolic blood pressure and systolic blood pressure which have the highest environmental correlation (Supplementary Table 4). While all three methods have a similar oscillation based on which traits have no environmental correlation, HIPO does not seem to respond as strongly as PAT and MTAG. Interestingly, when there was environmental correlation between B,H,S but not D, there was similar power to when there was no sample overlap (None). While the increase in power was expected, the increase was higher than when there was environmental correlation between B,D,H and no environmental correlation with S. Both of these scenarios only had one trait with no environmental correlation and removed the strongest source of environmental correlation between D and S. The reason for this larger increase when there was no sample overlap with D and not with S was because the second largest source of environmental correlation is between D and B. [pg. 26-27 and Supplementary Fig 1]

We also reference these simulations in the Results:

To better understand the effect of environmental correlation on statistical power, we conducted further simulations in the supplementary materials (see Supplementary Fig 1). [pg. 9]

- If I interpreted it correctly then the authors are determining statistical significance in HIPO if any component passes the threshold. However, with eigenvectors, the signal can get spread out across multiple components. I suspect this is happening in the simulations/real data because height is negatively correlated with the other traits. Can the authors combine the p-values across these components and then generate an empirical null similar to PAT to estimate empirical p-values. An initial thought could be to combine via Fisher's method and then probit-transform this p-value into a z-value. You can then estimate the empirical null for this statistic in a similar way as PAT. This is probably a better comparison to test the global null that leverages all the information across the components.

We thank the reviewer for bringing this to our attention. We set the significance threshold for HIPO and MTAG the way it was set in their respective papers. The idea of estimating an empirical null for HIPO is a very clever idea, but we feel it is beyond the scope of this paper to design and vet a novel extension to HIPO. We have, however, added a paragraph to the Discussion in order to promote the idea of null simulations as a means of calibrating the global null.

While we primarily considered how the methods handled the misspecification of the covariance structure between traits. Another fundamental difference between PAT and the other methods was how PAT derived its critical value using null simulations. In contrast, the other methods produced their p-values analytically; however, they could also leverage null simulations. This may be particularly beneficial to HIPO whose signal was likely spread across multiple components. Accounting for this empirically may better calibrate the global null. Further work would need to be done to explore this, but we note that importance sampling as used here would enable an efficient solution. [pg. 16]

- Shouldn't the estimated number of causal SNPs be different for each entry in the genetic covariance for the m-value optimization?

We thank the reviewer for bringing this to our attention. We acknowledge that the ground truth is each trait has a different number of causal SNPs; however, our intention here isn't to produce an accurate estimate of the number of causal SNPs for an individual trait, but to find the scaling of the genetic covariance matrix that best reflects the average effect size of the significant loci. As the original scaling for hypothesis testing in PAT assumes all SNPs are causal (the polygenic model), we thought of this rescaling in terms of estimating the number of causal variants. The simplest solution would have been to rescale just using the number of significant variants. As some associated SNPs are identified solely because of linkage disequilibrium, we instead used maximum likelihood to find the best scaling. In future work, we could incorporate a better estimate of the number of causal SNPs both for each trait as well as for each pair of traits; we have added this idea to the Discussion:

One limitation to the m-value interpretation framework was how it estimated the number of causal variants for the genetic covariance matrix. Currently, for association testing the genetic covariance matrix was scaled according to the polygenic model (i.e. all variants were causal). Once variants were implicated as associated, we used grid search to find the genetic covariance matrix scaling that best reflected the average effect size of independent variants. This in effect was an approximation to the number of causal variants. Further work is merited to better estimate the number of causal variants for each trait as well as the number shared between traits. [pg. 17]

- Does the calculation of m-values have to take into account the selection process (i.e., only calculate m-values when $p < alpha$)? So the actual distribution in the equation is not Gaussian but instead a truncated Gaussian?

We thank the reviewer for bringing this question to our attention. The reviewer makes a good point that the actual distribution of the prior using only significant variants does not follow a Gaussian distribution. However, this is not relevant because the purpose of m-values is to interpret a specific locus that has a significant association. For the purposes of interpretation, utilizing the observed association signals with a Gaussian prior makes the most sense. If we used a truncated Gaussian, we would need to use the significant association signal twice, once for the truncation and once for the posterior estimation. If a locus does not have a significant association, that locus is not of interest to be interpreted since it is not clear if there is a significant association in the locus in the first place.

## Minor comments

- Can the authors also include the computational time for each method in Table 1?

We thank the reviewer for this suggestion. We have added the running time as the last row of Table 1 and added the following sentences to the Results:

The final row in Table 1 contains the running time for each method. Here, we see ASSET was significantly slower than the other three methods which were comparable to each other. PAT's efficient running time indicates that the use of importance sampling can enables a speed up comparable to deriving p-values analytically; the differences in compute time between PAT,

HIPO, and MTAG were likely due to other factors (e.g. MTAG does a number of sanity checks prior to analysis). [pg. 9]
- m-values are a simple and effective method to help determine important traits. The discussion in the main text about m-values/z-values and the corresponding threshold of 0.9 was a bit confusing. m-values are a Bayesian quantity and the threshold is somewhat arbitrary in practice? The quantity itself is just a measure of the strength of evidence? If so, I think it should be made a bit clearer (and potentially simplified) in the main text.

We thank the reviewer for bringing this to our attention. The review raises a good point that the m-values threshold is arbitrary and is based on our previous work. We have edited the m-values result section to be clearer and hopefully simpler. [pg. 6-7]

In the previous section, we provided an overview of how PAT provides a per variant omnibus p-value for the set of traits. Here, we used simulated data reflective of four real UK Biobank traits (see Supplementary Tables 1-4) to provide some intuition about m-values as well as highlight its accuracy. M-values were produced by enumerating over the set of configurations C = $\{0, 1\}^4$ which indicate which trait(s) have a genetic effect, \Sigma_g(c). We note that the configuration c = (1,1,1,1) indicates a genetic effect in all four traits (i.e. \Sigma_g(c) = \Sigma_g). For each configuration, we calculated the posterior probability P(S |\mu = 0, \Sigma = \Sigma_e + \Sigma_g(c)). We then take the sum of the configurations compatible with trait i (c_i = 1) and divide by the total probability over all configurations to produce the m-value for trait i. If this ratio m_i > 0.9, we interpreted the omnibus variant-trait association to be an association between the variant and trait i. If this ratio m_i ≤ 0.9, we left the interpretation as ambiguous. We note that m-values are a Bayesian quantity whose threshold is a matter of convention established in previous work; in Supplementary Fig 2, we gave some empirical support for this threshold as the m-value can be seen as a very conservative bound on the false assignment rate.

In Fig 3, we simulated one million variants under two model conditions. In sub-figure A, there was a genetic effect in all four traits: body mass index, diastolic blood pressure, height, and systolic blood pressure while in sub-figure B, we modeled a genetic effect in only body mass index and height. In Fig 3, the truly associated traits were denoted with an asterisks (*) around the trait name. The effect sizes were simulated such that the first model has 50% power and 44% when there was a genetic effect in only body mass index and height. We split the summary statistics (z-scores) for each trait based on whether there was even modest signal in a particular trait (|z-score| > 3). This distinction was due to differing expectations on the ability to correctly interpret an association. We note that the inclusion of variants with a |z-score| ≤ 3 for a particular trait was primarily done for completeness and their interpretation was overwhelmingly ambiguous (Fig 3 right panel). We therefore, focus on the left panel of Fig 3 where the |z-score| > 3.

When there was a genetic effect in all four traits (top row left side), the m-value was greater than 0.9 for the vast majority of z-scores which means the majority of variants were correctly interpreted as associated with all traits. Diastolic and systolic blood pressure had the most ambiguous associated variants with 3,381 and 2,959, respectively. This, however, was still less than 2% of the variants with at least a modest effect size (|z-score| > 3) being interpreted as ambiguous for each trait. Furthermore, when there was a modest effect size the overall false negative rate was 0.6% across the traits.

The second set of simulations modeled a genetic effect in only two of the traits (bottom row left side). Here, the m-value framework correctly interpreted when there was a genetic effect in body mass index and height for most significant variants. For body mass index only 169 of the variants were missed and none were left ambiguous for height. For diastolic blood pressure and systolic blood pressure, approximately 1,200 variants for each trait that had a |z-score| > 3.

The m-value wrongly identified 951 and 1,019 of those variants as associated for diastolic blood pressure and systolic blood pressure, respectively. Overall, 99.5% of the variants analyzed for diastolic blood pressure and systolic blood pressure were left with an ambiguous interpretation. For body mass index and height, 64.5% and 90.5% of all variants, respectively, were correctly interpreted as associated when there was only a genetic effect in these two traits.

These simulations show the m-value framework has a low false positive assignment rate, and enabled the correct classification of many associated variants. This was especially true when |z-score| > 3 while |z-score| ≤ 3 typically resulted in the interpretation being ambiguous regardless of the ground truth. While this was still a false negative assignment, many of these associations would have failed to pass a nominal test for significance (p-value < 0.05). [pg 6-7]

- It would be useful to add a couple sentences in the methods section about choosing r for the importance sampling algorithm (and then reference the section in the supplement).

We thank the reviewer for pointing this out, we have added the following sentences to the methods section about importance sampling.

When picking the scaling factor r, larger values will sample the tail in fewer simulations, however, care should be taken to ensure that a sufficient number of simulations are used to accurately set the critical threshold \kappa. In Supplementary Table 6, we found that $10^6$ consistently provided a stable estimate of the critical value for \alpha = $5x10^{-8}$ regardless of the choice of r. [pg. 20]

## Typos

We thank the reviewers for taking the time to point these out to us, we have fixed the following mistakes and carefully reread the paper multiple times in hopes of preventing further typos.

- Double check the equations: there are small math mode typos across document (e.g., "max", "causal" not in text mode while in math environment)
- Page 16: In the regression model, e is the random error not the residual.
- Figure 2: Might want “s_1”, “s_2” to be in math mode. Also, might want to reorder the labels on figure legend so “No method” is on the bottom/top.
- Page 7/33: Supplementary Fig 3. -> Fig in main text
- Page 2: “Fiallly,” -> “Finally”

Reviewer #2:
The authors presented a powerful new method for multi-trait genetic association analysis, and a modified m-value for per-trait interpretation. In general, I found the method interesting, and the paper clearly written. However, I have the following comments:

-Table 2 compared the number of per-trait associations discovered by PAT and HIPO using m-values and MTAG using p-values. What is the p-value threshold for MTAG? Since p-values are not directly comparable to m-values, the authors need to justify the choice of p-value that is comparable to m-value>0.9. I would also suggest using precision-recall curve to compare the three methods, which is free of the effects of threshold choice.

We thank the reviewer for bringing this to our attention. Ultimately, as you have pointed out, the issue is p-values and m-values are not comparable. While a precision-recall curve would be free from the effect of a threshold choice in MTAG, it would still be an issue for PAT and HIPO since we only interpret genome-wide significant associations. We chose $5x10^{-8}$ as the p-value threshold for MTAG because it is the same threshold we used for association testing for PAT and HIPO. In the Supplementary Materials, we compare the methods more fairly, but this was stated at the end of the Results section. We have reorganized the section to state this clarification sooner and emphasize that the inclusion of MTAG (and ASSET) is rather for completeness as opposed to a fair comparison of fundamentally different concepts.

The four multi-trait methods were previously compared in regards to their power to perform omnibus association testing (see above). Here, we investigated the per trait interpretation of these associations. As MTAG computes a p-value for every trait, the method provides a direct per trait interpretation; therefore, for each respective trait we reported the variants with a p-value $< 5x10^{-8}$. The method, ASSET, considers all possible subsets and selects the one that maximizes its test statistic. This is done separately in the positive and negative directions of effect and are then combined for a two-tailed test which determines the omnibus association. For the associated variants, we tested each direction separately for significance (p-value $< 5x10^{-8}$ and interpreted the subsets that produced a significant association as the trait(s) driving the association. The last two methods, HIPO and PAT, only provided an omnibus interpretation; therefore, we applied the m-value framework to assign a per trait association to variants whose omnibus p-value $< 5x10^{-8}$. For both methods, this was done by taking the associated variants and calculating the posterior predictive probability (m-value) of whether there was a genetic effect in each particular trait. If the m-value was greater than 0.9, the variant was deemed associated with the trait. Otherwise, the interpretation was left ambiguous.

Prior to exploring the per trait interpretation, we note that only MTAG controls the false positive per trait interpretation due to its use of p-values for the assignment; m-values do not directly control for false positives. As a result, m-values are only meant to provide empirical insights and interpretation to p-values not replace them. This means the comparisons in Table 2 between MTAG's p-values and the m-value interpretations are not an apples to apples comparison. In Supplementary Fig 3, we provide a fairer comparison by ranking the p-values and m-values. There we show that for any false positive rate, PAT and HIPO have more true positive per trait assignments than MTAG. Separately, we acknowledge that while ASSET provides the subset of traits with the strongest association signal with the intent of a more interpretable multi-trait association. It is possible that a trait was included in the optimal subset

due to its tagging the causal signal in another trait. In this case, including the trait was useful for increasing the association power but would lead to an incorrect interpretation. [pg 10-11]

-I found the third column of Figure 2 a bit confusing for comparing MI GWAS and PAT, mainly because the density of dots is not well reflected and hence performance is not proportional by the area of each color. I suggest adding the proportion of dots falling into each color. It may also be helpful to use a smoothed density plot with color gradient proportional to density.

We thank the reviewer for pointing this out and we agree that this figure is confusing without the corresponding numbers. We updated the figure with larger axes and lower opacity to improve the figure. Additionally, we added Supplementary Table 7 which has exact counts in each region.

-Table 4 and page 14 shows low replication rate in the GIANT consortium. I would suggest including the replication rate of HIPO and MTAG to get a sense of the relative performance.

We thank the reviewer for this suggestion. We have added the replication of the HIPO and MTAG discoveries to the Results:

The three methods PAT, HIPO, and MTAG respectively identified 22,095, 19,829, and 931 novel associations when jointly analyzing four traits from the UK Biobank. For PAT, all novel associations had an m-value greater than 0.9 in at least one trait which means all associations had a per trait interpretation. The breakdown of the per trait associations were: 12,261 variants interpreted as associated with body mass index, 7,868 with diastolic blood pressure, 21,119 with height, and 7,605 were interpreted as associated with systolic blood pressure. For HIPO, there were 862 associations with an ambiguous interpretation. The breakdown of the 18,967 variants with a per trait interpretation were: 6,202 associated with body mass index, 8,420 with diastolic blood pressure, 6,396 with height, and 9,844 with systolic blood pressure. For MTAG which provided per trait p-values, 33 of the 931 novel associations were associated with body mass index, 254 with diastolic blood pressure, zero with height, and 644 were associated with systolic blood pressure. Now equipped with novel per trait associations, these discoveries should be validated in an external dataset; therefore, we used the GIANT consortium to see if any of the new associations for body mass index or height could be reproduced.

For body mass index, the European summary statistics from the GIANT consortium contained 2,554,638 variants which were separately matched to the variants identified by PAT, HIPO, and MTAG using the RSID, reference, and alternate allele and had a minor allele frequency reported. When the reference and alternate allele differed between the two data sets, the direction of the effect size in the replication data set was flipped. After identifying which variants were present in both data sets, the variants discovered by PAT and HIPO were clumped by taking the largest m-value (i.e., posterior predictive probability) and removing all other variants within a 1MB region. We clumped variants on the m-value instead of the p-value due to the p-value's significance potentially being driven by a different trait. For MTAG, as there was a per trait p-value, we clumped variants using the minimum p-value. Out of the 12,261 novel variants discovered by PAT and interpreted to be associated with body mass index in the UK Biobank, 3,946 were found in the GIANT consortium which resulted in 408 independent variants after clumping. Separately, for the 6,202 variants identified by HIPO, 2,111 were found in the GIANT consortium which resulted in 218 independent variants after clumping. Of the 33 novel variants discovered by MTAG, ten were found in the GIANT consortium which resulted in six independent variants after clumping.

This process was repeated in height where the GIANT consortium has 2,550,859 variants to be considered. Out of the 21,119 variants identified by PAT and interpreted as associated with height in the UK Biobank, there are 7,068 also found in the GIANT data set. After clumping these variants to the peak m-value per megabase region, there were 735 independent associations. Separately, for the 6,396 variants identified by HIPO, 2,216 were also in the GIANT consortium which resulted in 196 independent variants after clumping. MTAG identified zero novel variants associated with height.

In order to test the replication rate, we performed a one-sided z-test in the direction of the effect size (\beta) in the UK Biobank. Beginning with PAT, we saw that for body mass index, 378 out of 408 variants (92.6) had their effect sizes in the same direction in both cohorts. We tested each variant for replication using the level of significance \alpha = 0.05/408=$1.22x10^{-4}$ and found 14 variants replicated. For height, 97.4 (716) of the tested variants had their effect sizes in the same direction. For replication, we set the level of significance to \alpha = 0.05/735 = $6.80x10^{-5}$. Here, we saw that 197 of the 735 variants replicated. Separately, we considered the variants discovered by HIPO and saw that for body mass index, 209 of 218 (95.9) had their effect sizes in the same direction in both cohorts and 27 had a p-value below \alpha = 0.05/218 = $2.29x10^{-4}$. For height, 189 of the 196 (96.4) independent variants discovered by HIPO and interpreted as associated with height had effect sizes in the same direction in both cohorts and 43 had a p-value below \alpha = 0.05/196 = $2.55x10^{-4}$. For MTAG, the level of significance for body mass index was \alpha = 0.05/6 = $8.33x10^{-3}$. We observed two variants with a p-value below this threshold; all six variants (100) had their effect sizes in the same direction in both cohorts.

For the variants that failed to replicate, there were a number of possible reasons this occurred. In Table 4, we explored how statistical power affected our replication rate. We note that MTAG was not included in the table and that all six variants had a replication power over 90 with a mean of 97.2%. When considering the variants discovered by PAT and HIPO, we first binned the variants into deciles by their replication power. For each decile, we calculated the average power to replicate the effect sizes observed in the UK Biobank in the GIANT consortium. We note that the GIANT consortium did not release the minor allele frequency observed in their samples but instead provided the minor allele frequency observed in HapMap. While a reasonable estimate, inaccuracies in the minor allele frequency impact power calculations. Additionally, we note that the GIANT consortium summary statistics were from a meta-analysis which may have a lower effective sample size than the reported sample size due to heterogeneity between cohorts. For PAT, the average power over all variants for body mass index was 39.4% while it was 64.1% for HIPO, however we only saw a replication rate of 3.4% and 12.4%, respectively. For height, the replication rate for PAT was 26.8% and was 21.9% for HIPO, but the overall power was 65.5% and 47.3%, respectively. In Table 4, we observe for both traits that as power increased so did the replication rate; however, neither trait replicated at the expected rate. The only exception was in height when the power was between 0-30%, we saw PAT replicating slightly over the expected rate.

While we have shown that our replication rate was below expectation, the expected replication rate was likely overestimated due to winner's curse. As the variants tested for replication were not identified as associated by the original single trait GWAS, these variants have sub-optimal power for discovery. This means these variants have small effect sizes and were only found associated after leveraging their covariance structure with other traits. As a result, the bias in the effect size will be much larger here than in variants that were already well powered for discovery. For the non-replicating variants, further power increases (e.g. larger sample sizes) are essential to better tease out which variants warrant follow up analyses.

While many variants failed to replicate potentially due to insufficient power or winner's curse, we also tested whether the effect sizes were in the same direction between GIANT and the UK Biobank. If the variant truly had no effect on the trait, the concordance of effect size

across the data sets should be 50%; however, if there was a genetic effect, a higher concordance across data sets is expected. We performed a binomial test in each decile to determine whether the proportion of effect sizes in the same direction was greater than 0.50. Using the significance threshold \alpha = 0.05, every test was significant for PAT and 19/20 were significant for HIPO. As there were 20 tests, we adjusted for multiple testing using a Bonferroni correction (\alpha = 0.05/20). All but two tests were statistically significant at this new threshold for PAT and 15/20 were significant for HIPO. A test of overall concordance in body mass index tested whether 378/408 = 0.926 was greater than 0.5 returned a p-value of $4.34x10^{-78}$. We also tested height (716/735 = 0.974) which returned a p-value of $1.06x10^{-184}$. Separately, for HIPO, we tested the proportion of variants in the same direction in body mass index (209/2018 = 0.959; p=6.43x10^-51) and in height (189/196 = 0.964; p = $2.04x10^{-47}$). Therefore, we conclude that while the actual replication rate was low, there is evidence of real genetic signal in the variants identified by the multi-trait methods. [pg 13-15 and Table 4]

-The paper mainly uses the number of significantly associated variants as a metric of performance. This is okay in simulations, but in real data analysis (e.g. Table 3) it would be more interpretable to use the number of independent genetic loci.

We thank the reviewer for bringing this to our attention. The reviewer raises an interesting point about the number of independent loci being more interpretable. The issue is that two methods may identify the same locus with a different peak SNP which results in different clumping. If one of the methods finds a second signal in the set of SNPs pruned away by the other method by LD, is this a distinct signal? Separately, the peak SNP for each trait may differ in the locus, so the clumping of SNPs would depend on which trait is of interest; otherwise, for example, the clumping of PAT's omnibus signal is likely a proxy for clumping the signal in height. While contrived examples, we wanted to avoid inducing situations such as this, especially as different methods are better equipped to identify different variants. This is also why we clumped using m-values when we replicated to actually capture the distinct height/BMI signals to the best of our ability. With that in mind, we added the number of independent loci.

We note that these results were presented as the number of unique variants instead of independent loci implicated. This was done to better understand which set of variants each method identified as shown in Supplementary Table 5. (Otherwise, all variants in the locus would need to be truly associated with the same set of traits.) With that in mind, we report the number of unique loci identified genome-wide across traits. Single Trait GWAS returned the most independent loci (1,512) while the multi-trait methods, HIPO, MTAG, MI GWAS and PAT identified 1,340, 1,262, 1,347, and 1,324 respectively. [pg. 31]

In Tables 1 and 2, the definition of \sigma_g/causal is a bit confusing. If they refer to genetic effect size, why are they such large numbers? More description would be appreciated.

We thank the reviewer for pointing this out, and we acknowledge that the definition of \sigma_g/casual is confusing. We have changed the numerator to \Sigma_h2 in Table 1 and 2 to more clearly indicate that it is the genetic covariance matrix genome-wide. Under the polygenic model, the number of causal SNPs would be the total number of SNPs (e.g. 7.5 million). Instead of simulating under the polygenic model, we simulated the data with a covariance structure based on fewer causal variants. We could have chosen smaller numbers, this would have resulted in more statistical power across the board, but we worried about making the problem trivially easy for the methods. We added the following clarification to the Results.

For each of the configurations, there were three scaling factors for the heritability covariance matrix, \Sigma_h2, which can be thought of as the number of causal variants (\Sigma_h2/causal) where causal equals 40k, 24k, or 16k. We note that the methods assume the polygenic model (i.e. \Sigma_g = \Sigma_h2/# variants), but we simulated assuming fewer causal variants to create effect sizes large enough that there was power for discovery. [pg. 9]

Minor comments:
Page 2 fifth line from bottom: “finally” instead of “fially”.
Page 16 line 7 of section Generalizing GWAS testing to multiple traits (MI GWAS): “instead of” duplicated.

We thank the reviewer for finding these mistakes, we fixed these mistakes as well as read through our work very carefully to prevent further mistakes.

Reviewer #3:
The authors describe and address a pressing and common problem in genetic epidemiology: how to identify variants that (a) show evidence for pleiotropy (i.e. are associated with two or more traits) and (b) how to identify which traits a potentially pleiotropic variant is associated with. As the authors nicely review, there are many methods that leverage data on multiple traits to increase power to test the global null hypothesis that a variant is not associated with any of the studied traits--but these are not tests of pleiotropy per se (where the null is that the variant is associated with at most one trait), making it hard to assess whether more than one trait is contributing evidence for association or which traits are associated with the variant.

The authors propose a summary-statistic-based likelihood ratio test of the omnibus null. This test aims to use a decomposition of the cross-trait correlation into "environmental" and genetic components to improve power. They then propose "m-values" (an adaptation of their earlier work on GWAS meta-analyses) to identify which traits contribute to a significant omnibus test (and in particular whether more than one trait contributes). The use of summary statistics (as opposed to individual level data) is an important practical advantage. The proposed method appears to outperform the approaches they've chosen for comparison in many situations (both simulated and empirical).

The paper could use one more close read for grammar and typos, but I commend the authors on their clarity and organization. (As somebody who dabbles in this space, I'm often overwhelmed deciding which comparisons, which simulation scenarios, which data are worth highlighting.)

We thank the reviewers for noting that the paper is clear and well organized as well as the suggestions for additional methods to compare. We respond point by point below.

My main concerns/suggestions are:
1) Expanding (slightly!) the set of comparison methods

We thank the reviewers for bringing these methods to our attention. We have added ASSET to the Table 1 and 2 results where it performed quite well finding 3,820 associated variants which was better than HIPO and MTAG though m-values enable more per trait interpretations for HIPO than ASSET. We added these comparisons to the Results (but truncate the edits here as they span two sections and are integrated into the previous findings):

The final method is ASSET; this method works by searching for the subset of traits with the strongest positive signal and separately the strongest negative signal. ASSET then combines these test statistics using a chi-squared method to form an overall test statistic which we use for comparison… [pg 7-11, Table 1-2].

Separately, we added a section to the supplement which compared PAT’s rejection region to the rejection regions of MI GWAS, SUM, and VC.

In the main paper, we compared PAT and MI GWAS by simulating 100,00 summary statistics across four configurations of genetic and environmental correlation for two traits (see Fig 2). Here, we present these same simulations and included two additional methods: SUM and VC. SUM is a method designed to identify homogeneous effects amongst traits and computes a

weighted sum of z-scores using the between-trait correlation as the weight. The other method, VC (variance component), is better suited for discovering heterogeneous effects. Instead of modeling a fixed effect, the method assumes the effect in each trait is drawn centered around the overall mean effect with the covariance defined by the between-trait covariance.

In Supplementary Fig 4, each row corresponds to a different simulation framework and each method has its own column. The first row (sub-figures A-D), contains the simulations with 0% environmental correlation and 0% genetic correlation while the second row (sub-figures E-H) is when there is 0% environmental correlation and 67% genetic correlation. The third row (sub-figures I-L) contains the simulations with 67% environmental correlation and 0% genetic correlation while the fourth row (sub-figures M-P) has the simulations with 67% environmental correlation and 67% genetic correlation. For each simulation framework, we showed the rejection region of four multi-trait methods with each method in its own column and variants missed by the method in grey. In the first column, we present PAT and color variants discovered by the method as blue while in the second column, we present MI GWAS and color code the variants found as red. In the third column variants discovered by SUM are yellow, and the fourth column is VC and has the variants it discovered in green.

Here, we observed that the variance component methods: PAT and VC were elliptical in shape. MI GWAS as previously described (see Fig 2) was a square due to rejecting the null based on the trait with the minimum p-value while SUM was well powered in the direction of positive correlation and powerless when the correlation between traits was negative (e.g. x=-1, y=1). PAT was the only method whose rejection region changed with each adjustment to the genetic and environmental correlation. The other three methods only required knowledge of the null distribution (i.e. environmental correlation) to set their rejection regions; therefore, rows one and two have identical rejection region shapes and rows three and four have identical shapes for all methods except PAT.

We further explored these simulations by comparing the three methods: MI GWAS, SUM, and VC to PAT and present these results in Supplementary Fig 5 with the numerical values in Supplementary Table 7. Each row corresponds to one simulation framework and each column is a comparison between PAT and one other method. Variants discovered by both methods are shown in black, those missed by both methods are grey, and variants uniquely discovered by PAT are blue. In column one, we show a comparison between PAT and MI GWAS and is identical to the third column of Fig 2; variants discovered by MI GWAS are red. The second column compares PAT and SUM with the variants unique to SUM are yellow. The third column compares PAT and VC and has the variants unique to VC in green.

In Supplementary Fig 5, we saw that different rejection regions were better equipped to discover different variants. The first column was extensively discussed in the main paper, so here we focus on the second and third column. When we considered the comparison of PAT and SUM in the second column, we saw that PAT was more powerful when the summary statistics were negatively correlated; in this direction, SUM was powerless. When the summary statistics were positively correlated, SUM consistently discovered variants PAT failed to identify across simulation frameworks. Finally, in the third column we compared PAT and VC. These two methods were elliptical in shape and approximately identical in sub-figure C. In sub-figure F, PAT had more power in the direction of genetic correlation while VC had more power when summary statistics were negatively correlated which was similar to MI GWAS (sub-figure D).

However, when there was environmental correlation, as shown in rows three and four, MI GWAS was more powerful in the direction of environmental correlation and PAT was more powerful in the direction of negative correlation (sub-figures G and J). This was flipped from what was observed in sub-figure D. For VC, there was no flip in the direction of power. PAT was more powerful than VC in the direction of environmental correlation while VC was more powerful in the direction of negative correlation. While PAT and VC account for the environmental correlation in a similar manner, PAT also considered the genetic correlation which further shapes the rejection region which resulted in differing power between the methods. [pg 33-35, Supplementary Fig 4-5, Supplementary Table 7]

2) Being more clear in the main text of the impact of sample overlap across traits on the relative performance of methods

Thank you for this suggestion. We added simulations that model various environmental covariance structures by varying which traits have sample overlap. This was added to the Supplementary Materials (see Reviewer 1’s second comment).

We also added the following emphasis about PAT to the Results:

Additionally, we note that when there is no environmental correlation (\Sigma_e = I), PAT is comparable to a Wald test. [pg 3]

As for #1: First, there are relatively simple multi-trait test statistics that both account for the correlation in test statistics due to sample overlap and define the rejection region differently (arguably more cleverly) than MIPAT (see PMIDs 28653391 and 31564761). It might be interesting to include some these in the power comparisons and in the visual illustration of the test rejection region (Fig 2), especially the "SUM" and "variance component" tests (PMID 28653391).

Also, the ASSET method (PMID 22560090) is widely used as a multivariate test of association and as an ad hoc method for detecting pleiotropy/identifying which traits are associated with a variant (those classified as "positively" or "negatively" associated with the effect allele). It would be quite interesting to compare the power/sensitivity/specificity of ASSET to the other methods in Tables 1 and 2.

As for #2: the potential gain for the authors’ proposed likelihood ratio test—which decomposes the correlation in test statistics into an “environmental” and genetic component—relative to other methods—which do not decompose the test statistic correlation (like MI GWAS or MPATs 28653391 or ASSET)—seems to depend on the magnitude of sample overlap. No or very little sample overlap=no or very little “environmental”contributions, and PAT collapses to something like a Wald test (28653391). This needs to be emphasized more strongly and earlier. The authors’ simulations and applications focus on scenarios where there is substantial overlap. Worth presenting simulations or theoretical results for relevant scenarios where there is less overlap (eg GWAS meta-analyses of schizophrenia and bipolar disease; breast and ovarian cancer; breast and prostate cancer [!])?
