# Supplementary material for: Leveraging pleiotropy for joint analysis of genome-wide association studies with per trait interpretations
Source: PLoS Genet. 2022 Nov 7;18(11):e1010447. doi: 10.1371/journal.pgen.1010447 (PMC9671458; doi:10.1371/journal.pgen.1010447)
Supplement: Attachment — Submitted filename: ReviewerResponse_PAT_PLOSGentics.pdf [file pgen.1010447.s003.pdf]

UNIVERSITY OF CALIFORNIA, LOS ANGELES

BERKLEY • DAVIS • IRVINE • LOS ANGELES • MERCED • SANTA BARBARA
RIVERSIDE • SAN DIEGO • SAN FRANCISCO • SANTA CRUZ

September 13, 2022

Dear Dr. Epstein and Dr. Balding,

Thank you for considering our manuscript, “Leveraging pleiotropy for joint analysis of genome-wide association studies with per trait interpretations”, for publication in PLoS Genetics. We would like to thank the three reviewers for their comments and feedback. Following the suggestions of reviewer one, we have added an additional set of analyses considering positively correlated traits. We provide an inline response to the reviewer in red with new text in yellow highlight.

We thank the reviewers for their suggestions, as we believe these changes have improved the manuscript. We would also like to thank the editors again for considering our revised manuscript.

Sincerely,

Eleazar Eskin
Professor
University of California, Los Angeles

Reviewer #1: I thank the authors for the detailed responses to my comments.

- As mentioned in my previous review, it is worth exploring the role of the direction of genetic correlation (positive/negative) on PAT's power: As you mentioned in the text, the results in Table 1 suggest that PAT outperforms other methods when height is included (it has a negative genetic correlation with other traits) but otherwise it underperforms when all traits happen to have positive genetic correlations. Does PAT always underperform compared to the other procedures whenever the genetic correlation structure between traits are in same direction (e.g. all positive), or is this an artifact of the selected traits? I think this is important point to explore and discuss in the paper.

We thank the reviewer for their suggested further analyses, and we have added an additional set of analyses to the paper. We explored how the direction of effect impacts statistical power and the following text to the Supplementary Materials:

Through simulations, we showed that MTAG and HIPO were better powered to discover variants associated with diastolic and systolic blood pressure, and we hypothesized this was due to the strong environmental correlation between the two traits as it reflected the scenario in Fig. 1, where PAT was conservative in the direction of positive correlation. We were able to further support this in additional simulations shown in Supplementary Fig 1 by setting the environmental correlation between sets of traits to zero. In those simulations, we observed PAT had increased statistical power when there was no environmental correlation between diastolic and systolic blood pressure. In this section, we further explore the impact of environmental correlation by testing whether the correlation direction impacted statistical power. We compared PAT to MTAG and HIPO.

In Supplementary Table 5, we simulated seven configurations of environmental effect between two traits with 1 million simulations in each scenario. All simulation frameworks modeled a genetic effect in both traits and a genetic variance $\sigma_g^2=0.25$ for each trait but no genetic correlation. The sample size for each trait was $n=350,000$ individuals with complete sample overlap. All three methods were informed of the assumed genetic and environmental correlation structure. Here, we observed as environmental correlation increased to 50% as well as when it decreased negatively towards -50% MTAG and HIPO were better powered than PAT to discover associated variants. When there was moderate environmental correlation of 25% as well -25\% PAT, MTAG, and HIPO were approximately equally powered. As environmental correlation decreased to 10% and -10% as well as when there was 0% environmental correlation, we found that PAT was more powerful than MTAG and HIPO. We note that while the previous section showed that less environmental correlation reduced statistical power, this section indicated that more environmental correlation resulted in an increase in power for all three methods. The simulations here, however, do not have genetic correlation between the traits which dampens statistical power as shown in Supplementary Table 8. [pg 27-28]
